# Urinary cytokeratin 20 as a predictor for chronic kidney disease following acute kidney injury

**DOI:** 10.1172/jci.insight.180326

**Published:** 2024-05-28

**Authors:** Rui Ma, Han Ouyang, Shihong Meng, Jun Liu, Jianwei Tian, Nan Jia, Youhua Liu, Xin Xu, Xiaobing Yang, Fan Fan Hou

**Affiliations:** Division of Nephrology, Nanfang Hospital, Southern Medical University, National Clinical Research Center for Kidney Disease, State Key Laboratory of Organ Failure Research, Guangdong Province Institute of Nephrology, Guangdong Province Key Laboratory of Renal Failure Research Guangzhou, China.

**Keywords:** Nephrology, Chronic kidney disease, Cytoskeleton

## Abstract

**BACKGROUND:**

Identifying patients with acute kidney injury (AKI) at high risk of chronic kidney disease (CKD) progression remains a challenge.

**METHODS:**

Kidney transcriptome sequencing was applied to identify the top upregulated genes in mice with AKI. The product of the top-ranking gene was identified in tubular cells and urine in mouse and human AKI. Two cohorts of patients with prehospitalization estimated glomerular filtration rate (eGFR) ≥ 45 mL/min/1.73 m^2^ who survived over 90 days after AKI were used to derive and validate the predictive models. AKI-CKD progression was defined as eGFR < 60 mL/min/1.73 m^2^ and with minimum 25% reduction from baseline 90 days after AKI in patients with prehospitalization eGFR ≥ 60 mL/min/1.73 m^2^. AKI-advanced CKD was defined as eGFR < 30 mL/min/1.73 m^2^ 90 days after AKI in those with prehospitalization eGFR 45–59 mL/min/1.73 m^2^.

**RESULTS:**

Kidney cytokeratin 20 (CK20) was upregulated in injured proximal tubular cells and detectable in urine within 7 days after AKI. High concentrations of urinary CK20 (uCK20) were independently associated with the severity of histological AKI and the risk of AKI-CKD progression. In the Test set, the AUC of uCK20 for predicting AKI-CKD was 0.80, outperforming reported biomarkers for predicting AKI. Adding uCK20 to clinical variables improved the ability to predict AKI-CKD progression, with an AUC of 0.90, and improved the risk reclassification.

**CONCLUSION:**

These findings highlight uCK20 as a useful predictor for AKI-CKD progression and may provide a tool to identify patients at high risk of CKD following AKI.

**FUNDING:**

National Natural Science Foundation of China, National Key R&D Program of China, 111 Plan, Guangdong Key R&D Program

## Abstract 2

Cytokeratin 20 levels in urine are predictive of the progression of acute kidney injury to chronic kidney disease and may help identify patients at-risk early after injury.

## Introduction

Acute kidney injury (AKI) is a major global health problem with an annual incidence of 13 million cases worldwide (1) and is associated with increase in mortality and health care cost (2). AKI has long been considered a reversible condition. However, large population-based studies have demonstrated that patients who survive an episode of AKI are at considerable risk for progressing to chronic kidney disease (CKD) (3–5), a condition currently affecting 9% of the world’s population and responsible for 1.2 million annual deaths (6). To date, there is no pharmacological strategy to treat AKI or prevent disease progression from AKI to CKD. Studies to develop and evaluate the role of the prognostic markers for AKI to CKD progression would be of value and may ultimately guide monitoring of kidney function, appropriate follow-up, and selection of participants for clinical trials of novel interventions to prevent AKI to CKD progression.

Clinical and animal studies have demonstrated that the severity of AKI is a robust predictor for progression to CKD (7–9). Patients who have higher concentrations of serum creatinine or require dialysis are at especially high risk for progression to CKD (7). Furthermore, histologically diagnosed acute tubular injury (ATI) (8), particularly severe ATI, is often associated with progressive CKD (8–10). However, defining severe AKI by the currently used marker serum creatinine concentration has several limitations (10), and AKI is not frequently biopsied. Therefore, searching for a novel biomarker that can reflect the severity of ATI and be noninvasively measured may help physicians identify patients who are at high risk for AKI to CKD progression.

In this study, we established 2 types of mouse AKI model by kidney ischemia/reperfusion injury (IRI), i.e., mild AKI (kidney injury recovered after IRI) and severe AKI (kidney fibrosis occurred after IRI). By applying a kidney transcriptome-sequencing approach, we identified a noninvasive biomarker, cytokeratin 20 (CK20), for predicting AKI to CKD progression. In 2 cohorts of patients with AKI, we found that urinary concentrations of CK20 (uCK20) accurately predicted histologically severe ATI and AKI to CKD progression with excellent performance, particularly when added to an established clinical model (11). This finding may provide a tool to identify patients at high risk of CKD early following AKI.

## Results

### Identification of a candidate biomarker for predicting AKI to CKD progression

As the first step, we developed 2 types of IRI mouse model, mild (20-minute ischemia) and severe (40-minute ischemia) AKI. Although both models had increased serum creatinine within the first 3 days after IRI, tubular injury and renal function recovered within 7 days after IRI in mild AKI. In severe AKI, the histological tubular injury persisted and was followed by tubulointerstitial fibrosis 14 days after IRI. Similarly, subsequent tubulointerstitial fibrosis also was observed 14 days after the folic acid (FA) injection model of AKI (Supplemental Figure 1; supplemental material available online with this article; https://doi.org/10.1172/jci.insight.180326DS1).

**Figure 1:**
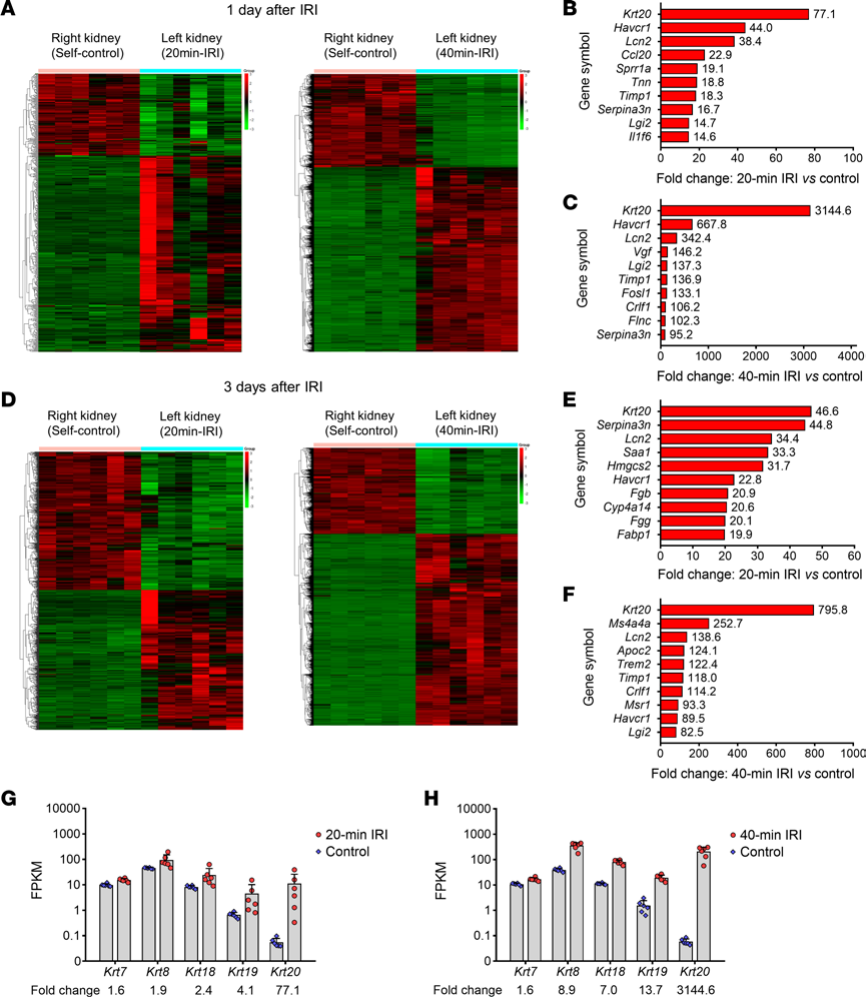
Cluster heatmap of expression profiles for differentially expressed genes in IRI mice with 20-minute or 40-minute ischemia. (**A**–**C**) Cluster heatmap (**A**) and top 10 upregulated genes between 20-minute IRI (left kidney) and self-control kidneys (right kidney) (**B**) or between 40-minute IRI and self-control kidneys (**C**) at 1 day after AKI. (**D**–**F**) Cluster heatmap (**D**) and top 10 upregulated genes between 20-minute IRI and self-control kidneys (**E**) or between 40-minute IRI and self-control kidneys (**F**) at 3 days after AKI. (**G**) Fold-changes in kidney fragments per kilobase of exon model per million mapped fragments (FPKM) between 20-minute IRI and self-control at 1 day after AKI. (**H**) Fold-changes in kidney FPKM between 40-minute IRI and self-control at 1 day after AKI.

To identify the markers for predicting AKI to CKD progression, we applied a transcriptome-driven sequencing strategy to screen the upregulated genes in the 2 IRI models. As shown in Figure 1, A–F, the top upregulated gene was *Krt20* (gene encoding CK20) in both mild and severe IRI models within 3 days after an episode of IRI. Compared with mild AKI, severe AKI had a 42-fold and 25-fold upregulation in *Krt20* in the kidney cortex at the first and third days, respectively, after IRI. Fragments per kilobase of exon model per million mapped fragments (FPKM) analysis showed that the upregulation levels of *Krt20* were remarkably higher than those of other reported kidney cytokeratin genes (Figure 1, G and H).

**Figure 2:**
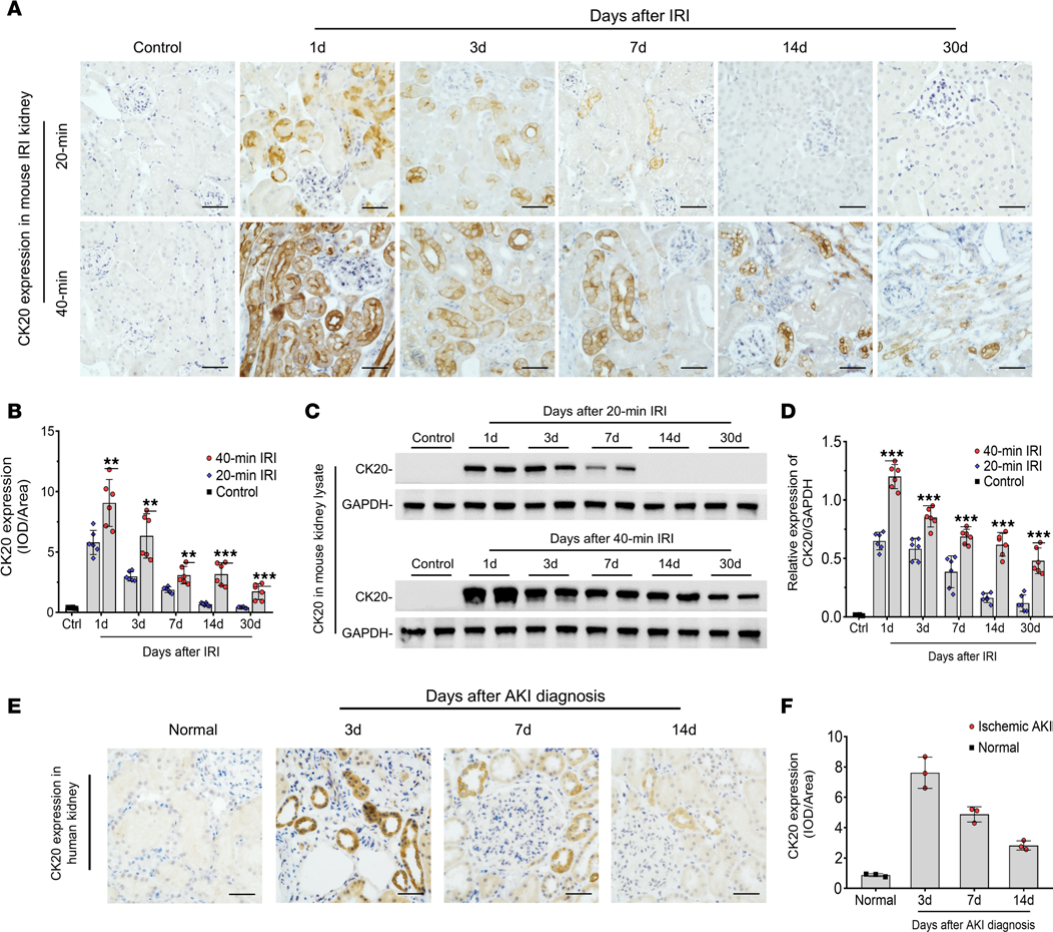
Upregulation of CK20 protein expression in kidney in severe mouse and human AKI. (**A**) Representative images of immunohistochemistry staining of CK20 in 20-minute and 40-minute IRI mouse kidneys at indicated times after AKI. (**B**) Semiquantitative analysis of integrated optical density (IOD)/area for **A**. (**C** and **D**) Western blotting (**C**) and semiquantitative data of CK20 (**D**) in kidney lysate after 20-minute and 40-minute IRI. (**E** and **F**) CK20 expression in kidney tissues was tested in 9 patients with ischemic acute tubular necrosis who underwent a kidney biopsy. Representative images of CK20 (**E**) and semiquantitative data (**F**) are shown. Normal kidney tissues adjacent to carcinoma obtained from surgery. *n* = 6 for each group of mice; *n* = 3 for each group of patients. Data are expressed as the mean ± SD. **P < 0.01, and ***P < 0.001. One-way ANOVA. Scale bar = 50 μm.

**Figure 3:**
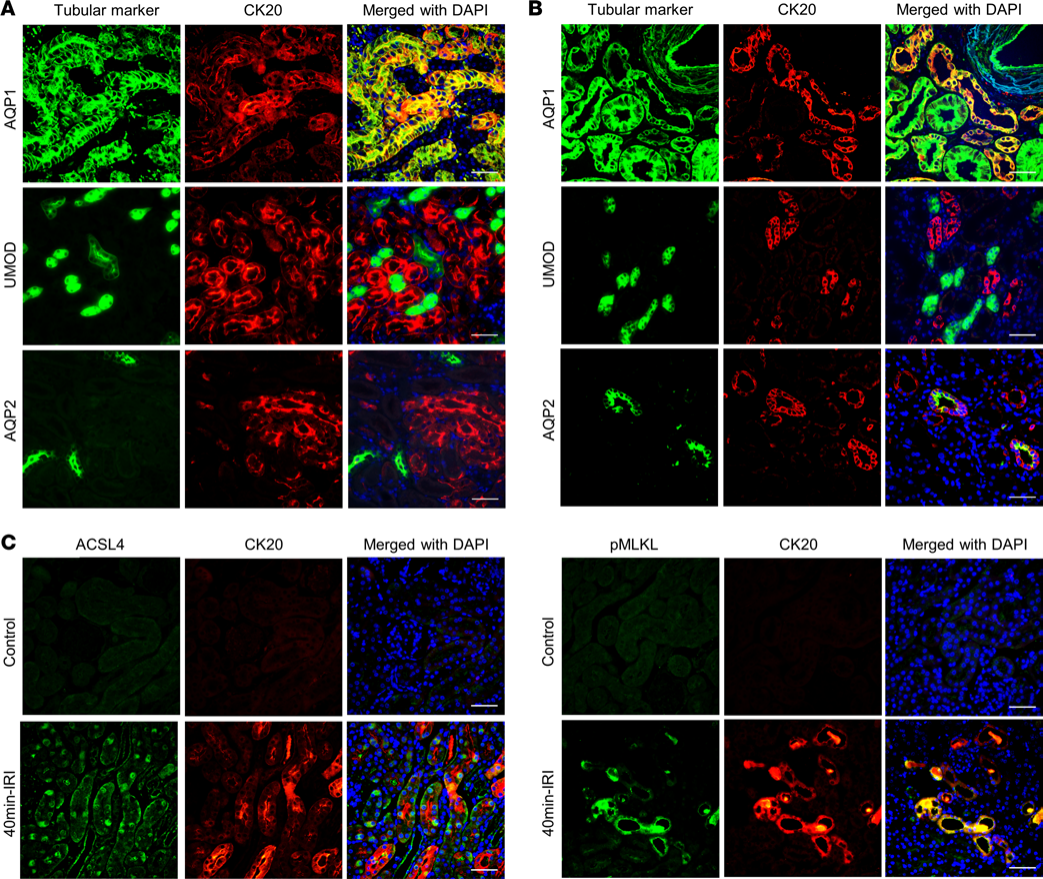
CK20 colocalizes with necrotic proximal tubular epithelial cells in IRI mice and human ischemic AKI. (**A**) Double-immunofluorescence staining to determine expression of CK20 and segment-specific tubular markers in kidneys from 40-minute IRI mice. The expression of CK20 colocalized with proximal tubule marker aquaporin 1 (AQP1) but not with thick ascending limb marker uromodulin (UMOD) and collecting duct marker aquaporin 2 (AQP2). (**B**) Double-immunofluorescence staining showed that CK20 was also expressed in proximal tubular epithelial cells in patients with ischemic AKI. (**C**) The expression of CK20 colocalized with necrotic tubular epithelial cells labeled by markers of ferroptosis (acyl-CoA synthetase long-chain family member 4, ACSL4) and necroptosis (phosphorylated mixed lineage kinase domain-like protein, p-MLKL). Scale bar = 50 μm.

**Figure 4:**
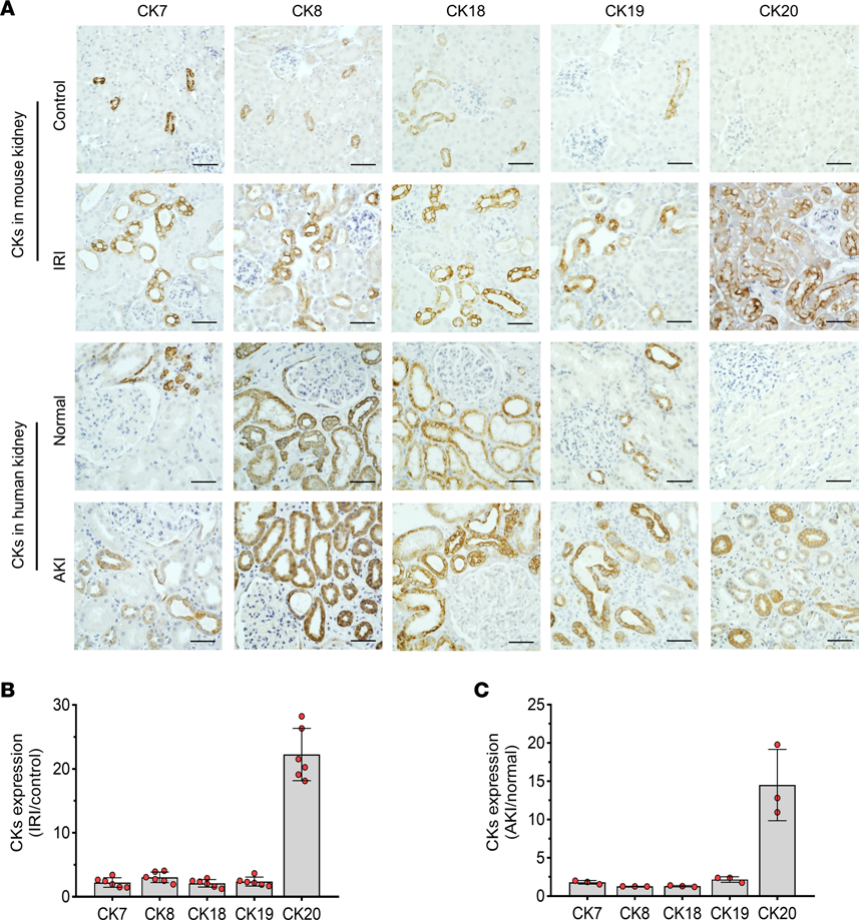
Upregulation of CK20 is the most remarkable among kidney cytokeratins in ATI. (**A**) Representative images of immunohistochemistry staining for kidney cytokeratins in mouse and human kidneys with or without ATI. (**B** and **C**) Upregulation levels of cytokeratins in mouse (ratio of IRI/control) (**B**) and human (ratio of AKI/normal) (**C**). ATI kidney samples were obtained from 40-minute IRI mice at day 1 or human ischemic ATI at day 7. *n* = 6 for each group of mice; *n* = 3 for each group of patients. Data are expressed as the mean ± SD. Scale bar = 50 μm.

Next, we identified the expression of the protein encoded by *Krt20*, CK20, in the kidney cortex (Figure 2). The expression of kidney CK20 was not detectable in any tubular segment in normal kidneys. However, upregulation of kidney CK20 was observed in the 40-minute IRI model, beginning at the first day and persisting to 30 days. In 20-minute IRI mice, CK20 expression was much lower compared with 40-minute IRI on the first day and undetectable at 14 days (Figure 2, A–D). Upregulation of CK20 was also observed in human severe ischemic AKI (Figure 2, E and F). Double-immunofluorescence staining showed that kidney CK20 was predominately expressed in proximal tubular epithelial cells in both mouse and human AKI (Figure 3, A and B). The expression of CK20 colocalized with necrotic tubular epithelial cells labeled by ferroptosis marker Acyl-CoA synthetase long-chain family member 4 (ACSL4) and necroptosis marker phosphorylated mixed lineage kinase domain-like protein (p-MLKL) (Figure 3C). The upregulation level of CK20 was the most remarkable among previously reported kidney cytokeratins (CK7, CK8, CK18, CK19) in AKI (12) (Figure 4, A–C).

**Figure 5:**
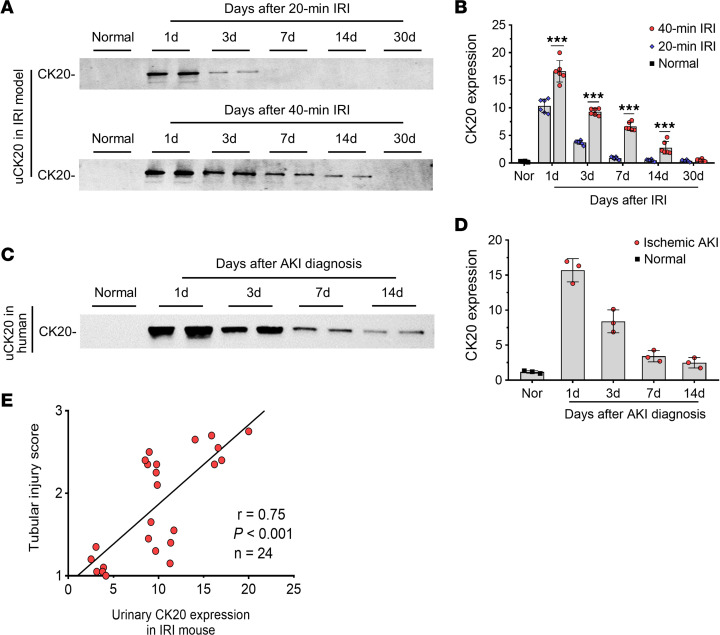
The levels of urinary CK20 increase in IRI mice and patients with ischemic AKI. (**A** and **B**) Western blotting (**A**) and semiquantitative data (**B**) of uCK20 at indicated times in 20-minute and 40-minute IRI mice. (**C** and **D**) Western blotting (**C**) and semiquantitative data (**D**) of uCK20 in patients with ischemic AKI. (**E**) Linear regression showed concentrations of uCK20 correlated with tubular injury scores within 3 days after IRI. *n* = 6 for each group of mice; *n* = 3 for each group of patients. Data are expressed as the mean ± SD. ***P < 0.001. One-way ANOVA.

Then, we detected uCK20 in mouse and human AKI to evaluate whether uCK20 can be used as a noninvasive biomarker. CK20 was not detectable in urine from healthy mice or humans. In mice with mild AKI, the increased concentrations of uCK20 decreased within 3 days after IRI (Figure 5, A and B). However, in mice or patients with severe AKI, upregulation of uCK20 persistently existed to 14 days (Figure 5, C and D). Level of uCK20 was associated with histological tubular injury score in IRI mice (*r* = 0.75, *P* < 0.001) (Figure 5E). Similarly, the upregulated expression of CK20 in kidney and urine was validated in the FA injection model of AKI (Supplemental Figure 2).

### Validation of uCK20 for predicting AKI to CKD progression in human AKI

#### Cohort characteristics.

After excluding patients without preadmission estimated glomerular filtration rate (eGFR), with preadmission eGFR < 45 mL/min/1.73 m^2^, who died within 90 days after AKI, or who were lost to follow-up, 204 survivors were enrolled in Test set, and 152 patients were included in Validation set (Supplemental Figure 3). The characteristics of the study cohort are shown in Table 1 (Test set) and Supplemental Table 1 (Validation set). AKI to CKD or advanced CKD progression was more frequently observed in patients with older age and higher levels of serum creatinine and UACR at the time of AKI diagnosis. Patients with AKI to CKD progression had higher concentrations of uCK20 as compared with those who did not progress.

Concentrations of uCK20 in patients with preadmission eGFR 45–59 mL/min/1.73 m^2^ but without AKI were higher than those in healthy volunteers (mean ± SD 2.3 ± 0.2 μg/g creatinine versus 1.5 ± 0.1 μg/g creatinine, *P* < 0.01). Patients with severe AKI (stage 2 or 3) had higher levels of uCK20 as compared with those with mild AKI (stage 1). There was no significant difference in the levels of uCK20 among patients with different etiologies (13) of AKI (Supplemental Figure 4).

Plasma CK20 in healthy volunteers was 2.4 ± 1.4 ng/mL. Plasma CK20 levels did not differ between AKI to CKD progressors and nonprogressors (Table 1 and Supplemental Table 1).

#### Levels of uCK20 are associated with the risk of AKI to CKD or advanced CKD progression.

**Figure 6:**
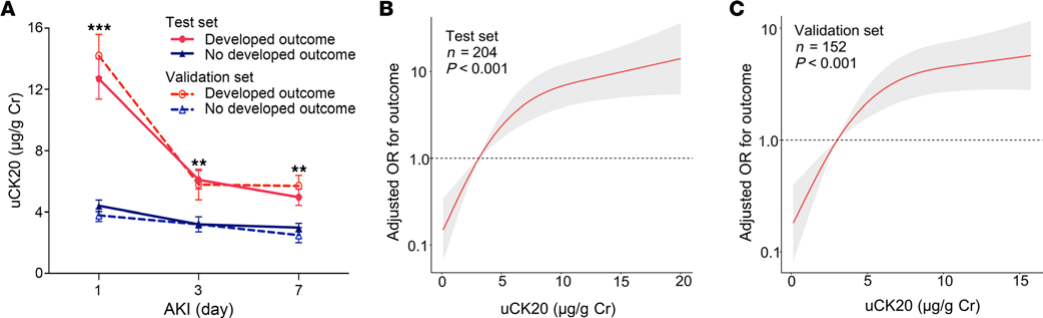
Association between uCK20 and the risk of AKI to CKD or advanced CKD progression in patients with AKI. (**A**) Dynamic changes in uCK20 within 7 days after AKI diagnosis. (**B** and **C**) Restricted cubic spline analysis between uCK20 at the time of AKI diagnosis and the adjusted OR of AKI to CKD or advanced CKD progression in the Test set (**B**) and Validation set (**C**). Analyses are adjusted for age, sex, baseline serum creatinine, UACR at the time of AKI diagnosis, and AKI stage. Data are expressed as the mean ± SD. **P < 0.01, and ***P < 0.001. Two-way ANOVA with Student-Newman-Keuls test.

Median time from AKI diagnosis to urine sample collection was 2 hours. Figure 6A displays dynamic changes in uCK20 over the first 7 days after AKI. Compared with patients who did not progress to CKD, those who progressed to CKD had a marked rise in uCK20 within 7 days after AKI, with the peak of uCK20 rise observed on the first day of AKI diagnosis. Restricted cubic spline displayed a positive association between uCK20 levels and risk of AKI to CKD or advanced CKD progression after controlling for age, sex, baseline serum creatinine, UACR at the time of AKI diagnosis, and AKI stage (Figure 6, B and C).

#### Levels of uCK20 are associated with the risk of histologically severe ATI.

In the Test cohort, 102 patients underwent a kidney biopsy in the Kidney Intensive Care Unit (KICU). The characteristics of this subgroup are shown in Supplemental Table 2. There were graded responses across the tertiles of uCK20 concentrations with histologically severe ATI in the unadjusted model (*P* < 0.05); the odds ratios (OR) remained statistically significant after adjusting for clinical variables. When uCK20 was analyzed as a continuous variable, higher levels of uCK20 were associated with severe ATI in the multivariable model (Supplemental Table 3).

#### Performance of uCK20 for predicting AKI to CKD or advanced CKD progression.

**Figure 7:**
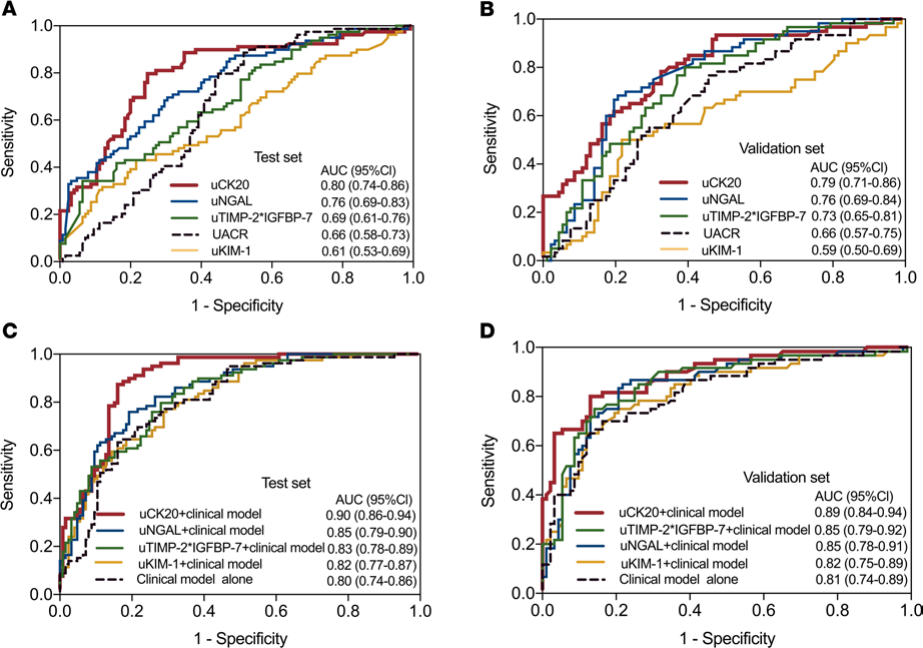
Performance of uCK20 alone or combined with a clinical model for predicting AKI to CKD or advanced CKD progression. (**A** and **B**) AUCs of uCK20 and other urinary injury biomarkers for predicting AKI to CKD or advanced CKD progression in the Test set (**A**) and Validation set (**B**). (**C** and **D**) AUCs of uCK20 or other kidney injury biomarkers combined with a clinical model for predicting AKI to CKD or advanced CKD progression in the Test set (**C**) and Validation set (**D**).

For predicting AKI to CKD or advanced CKD progression, the area under the receiver-operating characteristic (ROC) curve (AUC) of uCK20 in all participants was 0.80 (95% confidence interval [CI], 0.74–0.86) in the Test set. A cutoff of 5.0 μg/g creatinine yielded good sensitivity (0.80) and specificity (0.75) (Figure 7A and Supplemental Table 4). The AUC of uCK20 for predicting outcome was 0.79 (95% CI, 0.71–0.86) in the Validation set (Figure 7B and Supplemental Table 4). With addition of uCK20 to a clinical model comprising 6 clinical variables (age, sex, baseline serum creatinine, baseline UACR, severity of AKI, and serum creatinine level at discharge) (11), the AUC increased to 0.90 (95% CI, 0.86–0.94) for predicting the outcome in the Test set and 0.89 (95% CI, 0.84–0.94) in the Validation set, superior to other currently used biomarkers for predicting tubular injury or clinical model alone (Figure 7, C and D). For predicting histologically severe ATI by levels of uCK20, the AUC was 0.82 (95% CI, 0.74–0.90) (Supplemental Figure 5).

#### Improvement of risk reclassification with addition of uCK20 to the clinical model.

To determine whether uCK20 materially improved risk reclassification, we analyzed the net reclassification improvement (NRI) and the integrated discrimination improvement (IDI). As shown in Table 2, addition of uCK20 to the clinical model improved the risk reclassification of AKI to CKD or advanced CKD progression over combining the clinical model with other currently used urinary biomarkers for predicting tubular injury.

## Discussion

By applying kidney transcriptome sequencing for marker discovery, we identified a noninvasive prognostic biomarker, CK20, for AKI to CKD or advanced CKD progression. Expression of CK20 was upregulated in injured proximal tubular cells and detectable in urine within 7 days after an episode of AKI. Concentrations of uCK20 were independently associated with the severity of histological ATI and accurately predicted the risk of AKI to CKD or advanced CKD progression in patients with AKI due to various causes and at different stages.

AKI substantially increases the risk of consecutive CKD and its progression to advanced kidney disease (14). Identification of patients at risk of AKI to CKD progression is important but remains challenging. Several studies have been conducted to develop models for predicting the subsequent loss of kidney function following AKI (7, 9, 15–17). However, most of these models are limited to specific settings or designed to predict the risk of AKI requiring dialysis (11, 18). In this study of 204 patients with AKI, we found that uCK20 measured on the first day of AKI diagnosis was a powerful predictor for AKI to CKD or advanced CKD progression. High concentrations of uCK20 predicted consecutive CKD 90 days after AKI diagnosis. The performance of uCK20 was superior to currently used biomarkers for early detection of kidney injury, such as NGAL, KIM-1, and TIMP-2*IGFBP-7 (18), suggesting that uCK20 should be distinguished from previously reported kidney injury biomarkers. More importantly, adding uCK20 to an established predictive clinical model comprising 6 clinical variables (age, sex, baseline serum creatinine, baseline UACR, severity of AKI, and serum creatinine level at discharge) (11) improved performance of the model for predicting AKI to CKD progression with AUC from 0.80 to 0.90 and further improved the risk reclassification. These findings highlighted uCK20 as a useful predictor for AKI to CKD progression.

The mechanisms underlying the ongoing loss of renal function or incomplete recovery after an AKI episode are not completely understood. Tubular epithelial cells play a central role in inflammation and fibrosis following severe AKI (19, 20), thereby driving the progressive CKD. CK20 is a member of keratins, the intermediate filaments of the epithelial cell cytoskeleton with the most restricted expression pattern. The remarkable expression spectrum of CK20 among normal tissues comprises gastric foveolar epithelium, small intestinal epithelium, and certain neuroendocrine cells (21). The current knowledge about the function of CK20 is limited. The major kidney cytokeratins, CK7/CK19 and CK8/CK18, are expressed in the collecting ducts and CK8/CK18 in the glomerular parietal epithelial cells. These kidney cytokeratins are upregulated in stress situations, including IRI, with unknown biological importance (12). Our study found that expression of CK20 was not detectable in normal kidneys but upregulated in proximal tubular epithelial cells at an early stage of IRI. The expression level, at both gene and protein levels, was remarkably higher than that of other reported kidney cytokeratins. Consistent with our results, more recent studies report that progressive proximal tubular injury tracks with de novo activation of *Krt20* (gene encoding CK20) at 24 hours after IRI (22, 23). As an intracellular protein, CK20 might be leaked, not secreted, into urine when tubular epithelial cells experience destruction/necrosis during severe ATI. Colocalization of CK20 expression with markers of necrotic tubular epithelial cells supported this notion. Although this study was not able to interpret the functional meaning of CK20 overexpression during ATI, our pathologic analysis in human AKI further verified the activation of CK20 in injured proximal tubular epithelial cells. In particular, we identified uCK20 as a marker of the injury response that is independently associated with the severity of tubular damage (1 day after IRI) and after injury repair (7 days after IRI).

Current practice guidelines recommend that patients be followed up for 90 days to evaluate whether they developed CKD (24). However, because not all survivors from AKI progress to CKD (25–27), follow-up of all patients with AKI could lead to unnecessary use of clinical resources. Identifying patients at high risk of CKD progression early improved risk stratification and may improve the clinical outcomes in patients with AKI (28).

The strengths of this study include that the biomarker was identified by applying a hypothesis-free transcriptome-sequencing marker discovery approach. We tested the candidate biomarker uCK20 in a Test cohort and an independent Validation cohort of AKI due to various etiologies and at different stages. We compared predictive performance of uCK20 with other currently used biomarkers for early detection of kidney injury and verified its superiority. We further verified that adding uCK20 to an established clinical model improved risk reclassification for AKI to CKD or advanced CKD progression.

This study has several limitations. First, this is a “bench-to-bedside” study validated in a single-center cohort of patients with AKI. Further external validation is required. Second, the cohort of AKI used in the study included patients admitted to the KICU, with 73% of them having drug- and ischemia-induced AKI. More studies in patients with various AKI etiologies may be needed to further validate the marker. Third, the study did not investigate the changes in recovery/nonrecovery pathways at later times (after 30 days) of AKI, which may contribute to AKI to CKD transition (29).

In conclusion, we identified a noninvasive predictor of uCK20 for AKI to CKD or advanced CKD progression by applying a kidney transcriptome-sequencing marker discovery approach. In a 2-stage cohort of AKI due to various etiologies and at different stages, we found that concentrations of uCK20 accurately predicted histologically severe ATI and AKI to CKD or advanced CKD progression with excellent performance, particularly when added to an established clinical model. This finding may provide a tool to identify patients at high risk of CKD following AKI early.

## Methods

### Sex as a biological variable

Similar findings are reported for both sexes (22, 23), but our study examined male mice because male animals exhibited less variability in phenotype. Our cohort study included both men and women with AKI. All samples were pooled together for analysis, and sex was not considered as a biological variable.

### Study design

We used a 4-stage analysis to identify urinary biomarkers for predicting the risk of AKI to CKD progression. First, we used RNA sequencing to identify the top upregulated genes in the kidney of mice with tubular epithelial cell injury. Next, we determined whether the product of the top-ranking gene was upregulated in the tubular cells and its level correlated with ATI severity in mouse models and human kidney biopsy samples. Furthermore, we verified whether the product of the top ranked gene could be detected in urine of the patients with AKI and assessed the association between the urinary marker and the risk of AKI to CKD progression. Last, we tested the performance of the urinary marker, or its combination with the clinical model, for predicting AKI to CKD progression in 2 cohorts of patients with AKI.

### Mouse models

Male C57BL/6 mice aged 6–8 weeks (20–24 g) were purchased from Beijing SpePharm Biotechnology Co. Ltd. Mice were housed in a standard environment characterized by 12-hour light/12-hour dark cycle, 22°C–25°C, and 40%–60% humidity, with free access to water and forage.

IRI models were prepared as previously described (30, 31). Briefly, mice were anesthetized by sodium pentobarbital, and the right kidneys were removed as the self-controls. IRI was then induced by clamping the left renal pedicle for 20 minutes (mild AKI) or 40 minutes (severe AKI) by using microaneurysm clamps (Fine Science Tools company). Body temperature of mice was maintained at 37°C to 38°C by a temperature-controlled heating device during the process. Samples of kidney tissues, serum, and urine were harvested at the indicated times.

Models of FA-induced AKI were prepared by single intraperitoneal injection of FA (MilliporeSigma) at 250 mg/kg body weight, as described previously (32).

### Study cohort

We used data from a prospective 2-stage cohort. Patients with AKI who were admitted to the KICU were consecutively enrolled. The stage 1 study (Test set) was conducted in patients who were admitted to the KICU for AKI at Nanfang Hospital in China from February 2018 to January 2022. A total of 308 patients (18–80 years old) with AKI were screened. We excluded patients without preadmission eGFR, with preadmission eGFR < 45 mL/min/1.73 m^2^, who died within 90 days after AKI, and who were lost to follow-up. All survivors after hospital discharge were regularly followed up for at least 90 days. Data collection for the stage 2 study (Validation set) was also performed in patients with AKI who were admitted to the KICU at Nanfang Hospital from February 2022 to September 2023. A total of 179 patients were screened according to the same inclusion and exclusion criteria.

### mRNA sequencing in mouse kidneys

For mRNA sequencing, a brood of mice experienced left renal pedicle clamping for 20 minutes or 40 minutes, separately. Transcriptomics analysis was performed using renal cortex from left kidneys (injured kidneys) and right kidneys (self-controls). Concentration and integrity of RNA for each sample were assessed by Agilent 2100 Bioanalyzer. After mRNA enrichment, RNA-sequencing libraries were constructed by the Kapa Biosystems Standard RNA-seq Library Prep Kit and sequenced using Illumina X-ten/NovaSeq for 150 cycles. Raw sequencing reads were filtered using FastQC (version 0.11.7). Trimmed reads were aligned with the reference genome GRCm38 using Hisat2 (version 2.1.0). Gene expression levels were calculated using Ballgown (version 2.10.0) and normalized by library size and gene length into FPKM. To reduce transcription noise, each gene with FPKM below 0.01 was dropped for further analyses. The differentially expressed genes were identified with upregulated fold-change ≥ 2 and FDR-adjusted *P* < 0.05.

### Evaluating histologic tubular injury and fibrosis

Mouse kidney tissue was fixed in 10% formalin, embedded in paraffin, and cut into sections with 4 μm thickness for hematoxylin and eosin (HE) and Masson’s trichrome staining (MTS). Tubular injury was scored semiquantitatively on a scale of 0–3 as follows: 0, no tubular injury; 1, <25% tubules injured; 2, 25%–50% tubules injured; and 3, >50% tubules injured, as previously described (33). The degree of tubulointerstitial fibrosis was assessed by using ImageJ software (version 1.53t, NIH) in MTS-stained sections and expressed as the ratio of collagen deposition area over the whole cortical area.

Histological ATI in human kidney biopsy samples was detected in sections with 1 μm thickness and stained by HE and MTS as described above. The severity of ATI was defined as previously reported (34) and was categorized as: mild ATI, injured tubular involvement < 25%; severe ATI, injured tubular involvement 25%–50% or >50%.

### Expression of CK20

#### Immunohistochemical staining.

The mouse or human paraffin-embedded kidney sections were stained by using immunohistochemical or immunofluorescence staining, as previously described (35). Briefly, after being deparaffinized and rehydrated, sections received heat-induced antigen retrieval and blocking and were incubated with primary antibodies overnight at 4°C. The sections were detected by the EnVision/HRP Kit (Dako) or fluorescence dye conjugated with secondary antibodies. Human kidney tissues adjacent to carcinoma, obtained from surgery (*n* = 3), were used as the normal controls in immunohistochemical analysis.

#### Western blotting.

To assess the expression of CK20 in mouse kidney and urine and human urine samples, we applied Western blotting as previously reported (35). Briefly, mouse kidney tissues were lysed and proteins were extracted, then separated on an SDS-PAGE and transferred to a PVDF membranes (Merck Millipore). Membranes were blocked with 5% milk and incubated with primary antibodies overnight at 4°C. After reacting with HRP-conjugated secondary antibodies, the bands were visualized by a Minichemi chemiluminescence imager (Sage Creation Science).

#### ELISA analysis.

To analyze the levels of CK20 protein in human urine and plasma samples, an ELISA kit (Cusabio) was used according to the manufacturer’s instructions. The intra- and interassay variability were less than 8% and 10%, respectively. The lower limit of detection was 0.625 ng/mL. Urinary concentrations of CK20 were normalized to urinary creatinine and expressed as μg/g Cr.

### Measurement of urinary biomarkers for predicting tubular injury

Urine and plasma samples of study participants were collected within 24 hours after AKI diagnosis. We also took samples from age- and sex-matched healthy volunteers (*n* = 40) and patients with stage 3 CKD without AKI (*n* = 37) as controls. Urine samples were centrifuged at 3,000*g* for 10 minutes, and the supernatants were stored at –80°C. Previously reported urinary biomarkers for predicting tubular injury were measured using commercial ELISA kits according to the manufacturer’s instructions. Urinary albumin and creatinine were measured using a BA400 automatic analyzer (BioSystems). The biomarkers were measured by personnel masked to patients’ clinical data, including AKI status. The concentrations of urinary biomarkers were normalized to urinary creatinine and expressed as μg/g Cr.

All the antibodies and the ELISA kits used in this study are listed in Supplemental Table 5.

### Outcome and definitions

When available, baseline serum creatinine (for determining baseline eGFR) was defined as the most recent (within 1 month) level prior to admission (36). AKI was defined according to the Kidney Disease Improving Global Outcomes (KDIGO) Clinical Practice Guidelines for AKI (24), i.e., an increase in serum creatinine by 26.5 μmol/L (0.3 mg/dL) within 48 hours or a 50% increase in serum creatinine from the baseline level within 7 days. AKI stage was determined according to KDIGO guidelines. The eGFR was determined by the Chronic Kidney Disease Epidemiology Collaboration equation (37).

The outcome was AKI to CKD or advanced CKD progression. In patients with preadmission eGFR ≥ 60 mL/min/1.73 m^2^, AKI to CKD progression was defined as a persistent eGFR < 60 mL/min/1.73 m^2^ and with a minimum 25% reduction from baseline eGFR 90 days after AKI, which was confirmed by at least 2 separate measurements (38, 39). In patients with prehospitalization eGFR 45–59– mL/min/1.73 m^2^, AKI to advanced CKD was defined by a sustained reduction of eGFR < 30 mL/min/1.73 m^2^ 90 days after AKI.

### Statistics

Continuous variables were presented as mean ± SD or median (IQR). Categorical variables were presented as proportions. A 1-way ANOVA or a Mann-Whitney *U* test was used to test between-group differences for continuous variables, and the χ^2^ test was used for categorical variables. The change in uCK20 over 1, 3, and 7 days after AKI among patients with and without AKI to CKD progression was compared by 2-way ANOVA with Student-Newman-Keuls test.

The nonlinearity pattern of the association between uCK20 at time of AKI diagnosis and AKI to CKD or advanced CKD progression was checked by logistic regression using a restricted cubic spline adjusted for age, sex, baseline serum creatinine, UACR at time of AKI diagnosis, and AKI stage. The selection of the clinical variables for adjustment was based on patients’ characteristics and the risk factors of AKI development or nonrecovery reported in previous studies (11, 40). The association between uCK20 at the time of AKI and severe ATI was checked by logistic regression as well as by the tertiles and the logarithmic transformation of uCK20. The raw and adjusted OR and their corresponding 95% CIs were reported.

The discrimination performance of various risk prediction models for AKI to CKD or advanced CKD progression, including clinical factors alone, biomarker alone, and combination of risk factors (11) and biomarkers, was estimated and compared using the AUCs (41). The best cutoff values were determined by the Youden Index method. We also estimated the NRI and the IDI of the models including combination of clinical factors and biomarkers using the clinical model as the reference (42, 43).

All statistical analyses were performed using R version 4.1.2. Two-tailed *P* values less than 0.05 were considered statistically significant.

### Study approval

All animal studies were approved by the Nanfang Hospital Animal Care Committee (NFYY-2021-1043). The cohort study was approved by the Institutional Review Board of the National Clinical Research Center of Kidney Disease and the Medical Ethics Committee of the Nanfang Hospital, Southern Medical University (NFEC-2019-094). All the study participants provided written informed consent.

### Data availability

The kidney transcriptome-sequencing data are available at National Center for Biotechnology Gene Expression Omnibus database under accession number GSE266436. Values for all data points in graphs are reported in the Supporting Data Values file.

## Author contributions

FFH and XY designed the study. RM and HO performed most experiments and data analysis. SM and JL participated in the animal experiments. JT provided guidance with the experiments. NJ, RM, and HO performed pathological analysis and histological scoring. XX contributed to data and statistical analysis. FFH drafted the manuscript. YL gave advice on design and writing.

## Supplementary Material

Supplemental data

ICMJE disclosure forms

Unedited blot and gel images

Supporting data values

## Figures and Tables

**Table 1:**
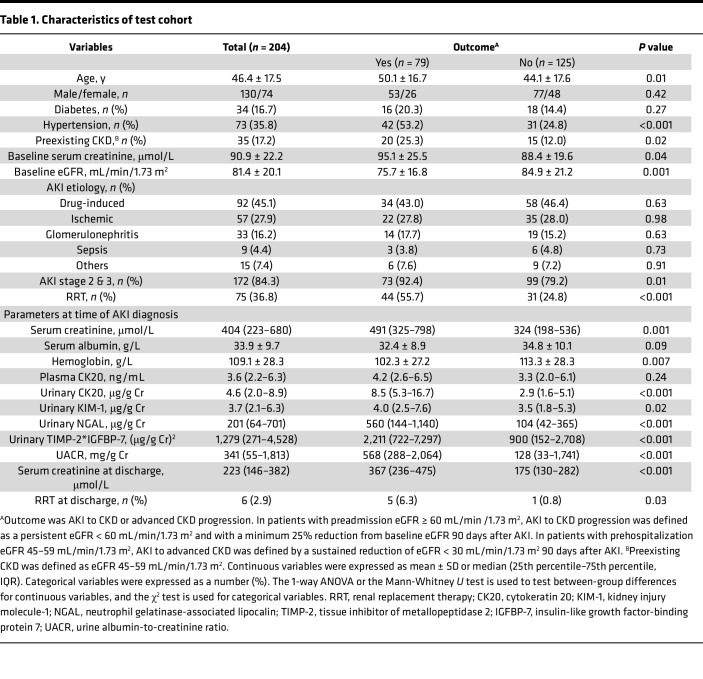
Characteristics of test cohort

**Table 2:**
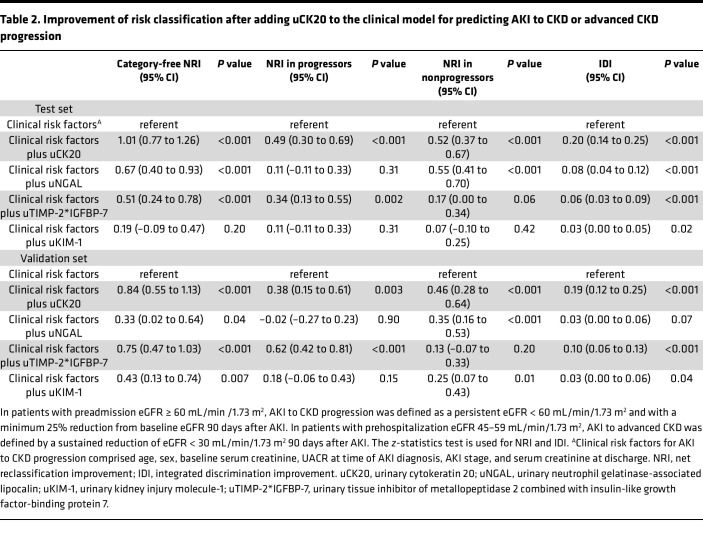
Improvement of risk classification after adding uCK20 to the clinical model for predicting AKI to CKD or advanced CKD progression

## Version 1 05/28/2024

In-Press Preview

## Version 2 07/08/2024

Electronic publication
