# Supplementary material for: Urinary cytokeratin 20 as a predictor for chronic kidney disease following acute kidney injury
Source: JCI Insight. 2024 May 28;9(13):e180326. doi: 10.1172/jci.insight.180326 (PMC11383368; doi:10.1172/jci.insight.180326)
Supplement: Supplemental data [file jciinsight-9-180326-s061.pdf]

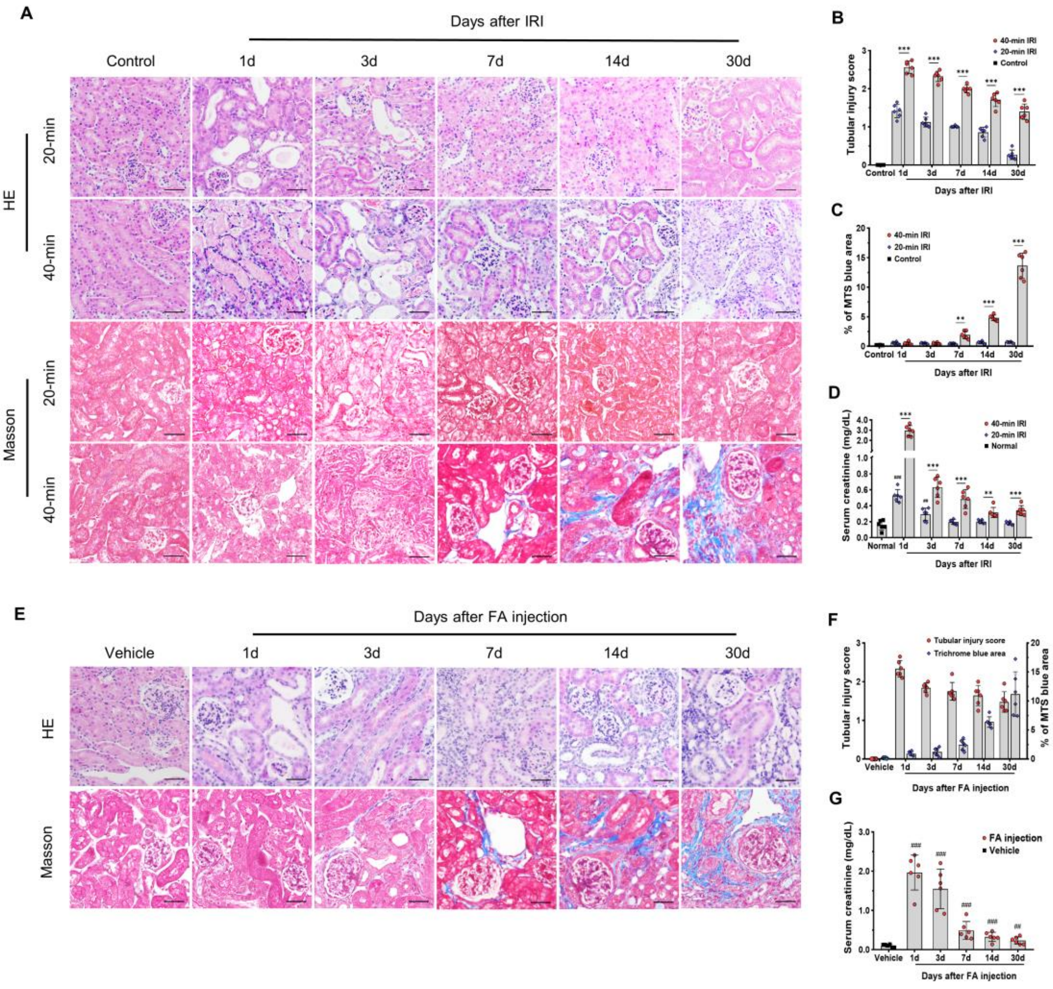

Supplemental Figure 1. Acute tubular injury, subsequent interstitial fibrosis, and changes in kidney function after IRI and FA-injection in mouse models.

(**A**) Representative images of HE and Masson's trichrome staining in mouse kidneys with 20-min and 40-min IRI at indicated time after AKI. (**B** and **C**) Tubular injury score based on HE staining (**B**) and the percentage of the positive area for Masson's trichrome staining (**C**) in 20-min and 40-min IRI kidneys. (**D**) Levels of serum creatinine at indicated time after 20-min and 40-min IRI. (**E**) Representative images of HE and Masson's trichrome staining on FA-injection mouse kidneys at indicated time. (**F**) Tubular injury score based on HE staining and the percentage of the positive area based on Masson's trichrome staining in FA-injection mice. (**G**) Levels of serum creatinine at indicated time in mouse after FA-injection. n = 6 for each group of mice. Data are expressed as the mean ± SD. *Comparison between 20-min and 40-min IRI, ***P* < 0.01, and ****P* < 0.001; #compared with control/normal/vehicle group, ##*P* < 0.01, and ###*P* < 0.001. Scale bar = 50μm.

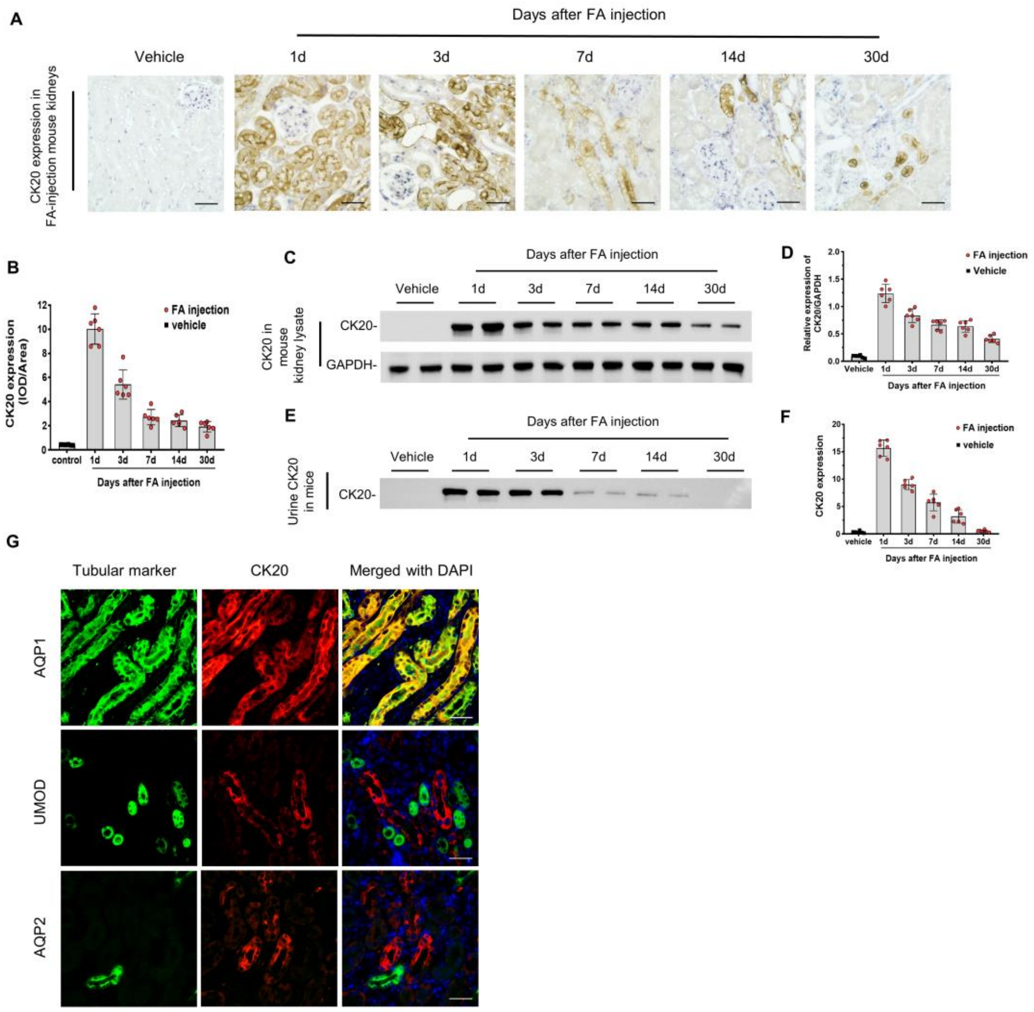

Supplemental Figure 2. Expression of CK20 in FA-injection mouse model.

(**A**) Representative images of immunohistochemistry staining of CK20 in FA-injected mouse kidneys at indicated time after AKI. (**B**) Semi-quantitative analysis of IOD for Figure A. (**C** and **D**) Western blotting (**C**) and semi-quantitative data (**D**) of CK20 levels in kidney lysate after FA-injection. (**E** and **F**) Western blotting (**E**) and semi-quantitative data (**F**) of uCK20 concentrations at the indicated time in FA-injection mice. (**G**) Double immunofluorescence staining to determine expression of CK20 and segment-specific tubular markers in kidneys from FA-injection mice. The expression of CK20 colocalized with proximal tubules marker AQP1, but not with thick ascending limbs marker UMOD and collecting ducts marker AQP2. Data are expressed as the mean ± SD. n = 6 for each group. Scale bar = 50μm.

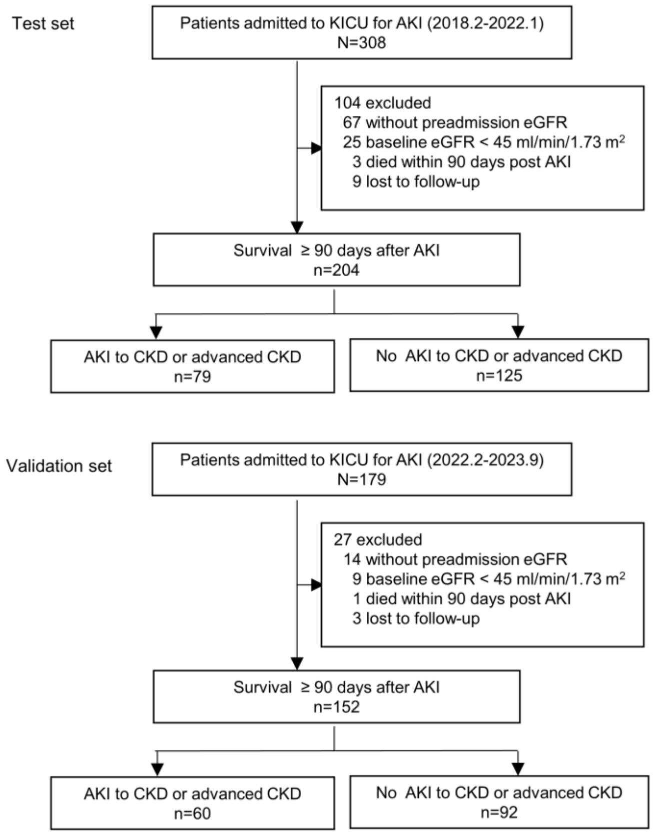

Supplemental Figure 3. Flow chart for Test and Validation set.

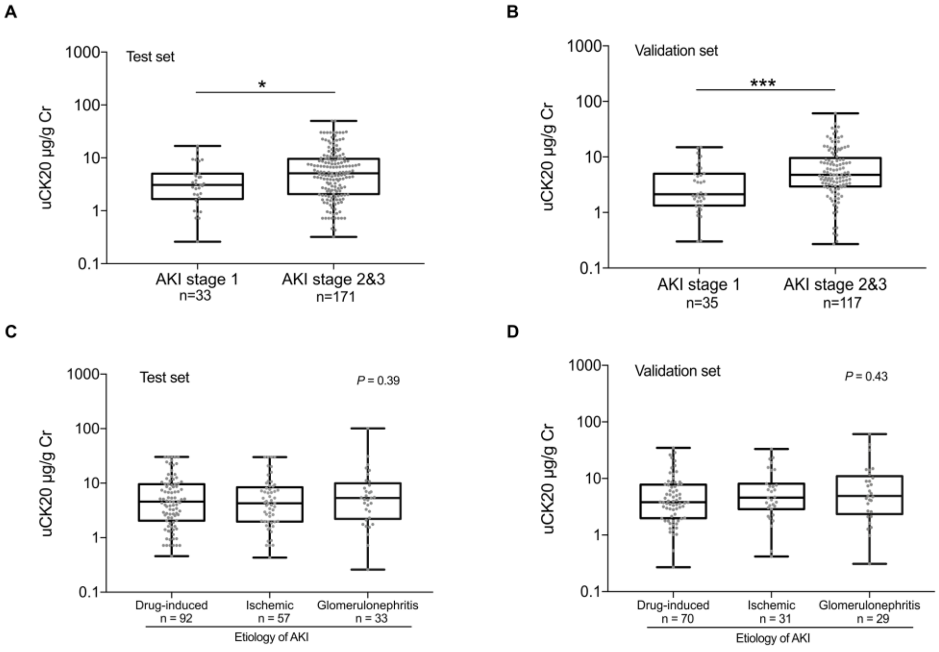

Supplemental Figure 4. Concentrations of uCK20 among patients grouped by AKI stages and etiologies in Test and Validation set.

(**A** and **B**) uCK20 concentrations in mild AKI (stage 1) and severe AKI (stage 2 or 3) in Test set (**A**) and Validation set (**B**). (**C** and **D**) uCK20 concentrations in different AKI etiologies in Test set (**C**) and Validation set (**D**). The horizontal lines represent the median and bounds of box-and-whisker plots representing the 25th and 75th percentiles, with upper and lower extremes represented by whiskers. Mann-Whitney test or Kruskal-Wallis test, *$P < 0.05$, and ***$P < 0.001$.

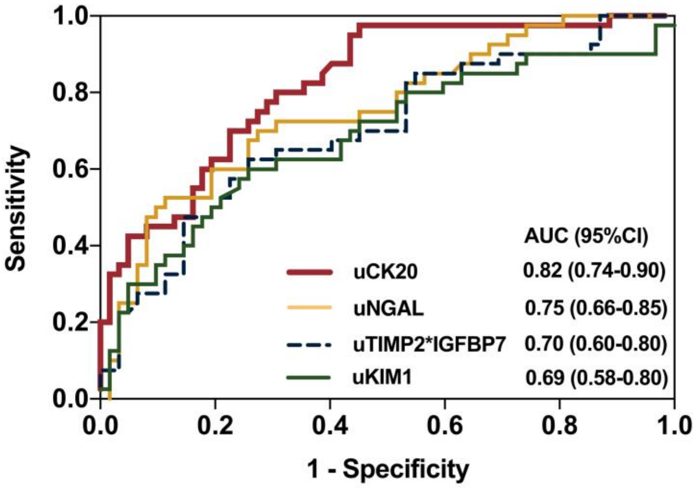

Predicting severe ATI in patients who underwent kidney biopsy

Marker	Best cutoff	Sensitivity	Specificity
uCK20, μg/g Cr	5.2	0.78	0.71
uNGAL, μg/g Cr	253.2	0.70	0.73
uTIMP-2*IGFBP-7 $(\mu g/g\ Cr)^2$	2143.6	0.63	0.74
uKIM-1, μg/g Cr	5.2	0.58	0.76

Supplemental Figure 5. AUCs of uCK20 and other kidney injury biomarkers for predicting severe ATI in patients received kidney biopsy.

Severe ATI was defined as injured tubular involvement > 25%. n=102 for patients received kidney biopsy.

Supplemental Table 1. Characteristics of Validation cohort

Variables	Total (n=152)	Outcome[A] Yes (n=60)	Outcome[A] No (n=92)	*P* value
Age, yr	44.2 ± 17.5	49.0 ± 17.4	41.1 ± 16.9	0.006
Male/female, n	107/45	40/20	67/25	0.41
Diabetes, n (%)	24 (14.8)	9 (20.3)	15 (11.8)	0.82
Hypertension, n (%)	63 (41.4)	31 (51.7)	32 (34.8)	0.04
Pre-existing CKD[B], n (%)	21 (13.8)	13 (21.7)	8 (8.7)	0.03
Baseline serum creatinine, μmol/L	92.6 ± 22.7	95.3 ± 25.7	90.9 ± 20.5	0.24
Baseline eGFR, ml/min/1.73m^2	82.8 ± 22.6	76.8 ± 20.3	86.7 ± 23.2	0.01
AKI etiology, n (%)				
Drug-induced	70 (46.1)	24 (40.0)	46 (50.0)	0.23
Ischemic	31 (20.4)	18 (30.0)	13 (14.1)	0.03
Glomerulonephritis	29 (19.1)	12 (20.0)	17 (18.5)	0.82
Sepsis	10 (6.6)	3 (5.0)	7 (7.6)	0.52
Others	12 (7.9)	3 (5.0)	9 (9.8)	0.28
AKI stage 2&3, n (%)	117 (77.0)	56 (93.3)	61 (66.3)	<0.001
RRT, n (%)	42 (27.6)	28 (46.7)	14 (15.2)	<0.001
Parameters at time of AKI diagnosis				
Serum creatinine, μmol/L	328 (188-580)	526 (320-737)	234 (159-438)	<0.001
Serum albumin, g/L	33.8 ± 8.4	32.7 ± 8.0	34.5 ± 8.6	0.18
Hemoglobin, g/L	108.1 ± 32.9	94.9 ± 24.7	116.6 ± 34.8	<0.001
Plasma CK20, ng/mL	3.0 (1.9-4.8)	3.4 (2.5-6.1)	2.9 (1.6-4.3)	0.09
Urinary CK20, μg/g Cr	4.2 (2.1-8.6)	7.7 (4.2-16.6)	3.0 (1.8-5.1)	<0.001
Urinary KIM-1, μg/g Cr	3.3 (1.8-5.0)	4.3 (1.8-6.2)	3.0 (1.7-4.3)	0.05
Urinary NGAL, μg/g Cr	148 (61-508)	402 (148-783)	106 (47-175)	<0.001
Urinary TIMP-2*IGFBP-7, (μg/g Cr)^2	1007 (166-3025)	1762 (999-4799)	504 (111-1557)	<0.001
UACR, mg/g Cr	541 (83-2359)	1135 (395-3148)	273 (55-1628)	<0.001
Serum creatinine at discharge, μmol/L	211 (156-357)	328 (217-502)	181 (133-232)	<0.001
RRT at discharge, n (%)	3 (2.0)	3 (5.0)	0 (0)	0.03

[A]Outcome was AKI to CKD or advanced CKD progression. In patients with preadmission eGFR ≥ 60 ml/min /1.73m^2, AKI to CKD progression was defined as a persistent eGFR < 60ml/min/1.73m^2 and with a minimum 25% reduction from baseline eGFR 90 days after AKI. In patients with prehospitalization eGFR 45-60ml/min/1.73m^2, AKI to advanced CKD was defined by a sustained reduction of eGFR < 30 ml/min/1.73m^2 90 days after AKI.

[B]Pre-existing CKD was defined as eGFR 45-60ml/min/1.73m^2.

Continuous variables were expressed as mean ± SD or median (25th percentile-75th percentile, interquartile range). Categorical variables were expressed as a number (%).

RRT, renal replacement therapy; CK20, cytokeratin 20; KIM-1, kidney injury molecule-1; NGAL, Neutrophil gelatinase-associated lipocalin; TIMP-2, tissue inhibitor of metallopeptidase 2; IGFBP-7, insulin-like growth factor-binding protein 7; UACR, urine albumin to creatinine ratio.

Supplemental Table 2. Characteristics of 102 patients who received kidney biopsy

Variable	Total (n=102)	Outcome[A]		*P* value
		Yes (n=40)	No (n=62)	
Age, yr	45.8 ± 17.1	51.7 ± 13.8	41.6 ± 18.1	0.02
Male/female, n	69/33	28/12	41/21	0.81
Baseline serum creatinine, µmol/L	84.4 ± 14.2	87.5 ± 12.8	82.2 ± 14.9	0.17
Baseline eGFR, ml/min/1.73m^2	85.7 ± 17.4	79.3 ± 9.2	89.8 ± 20.0	0.002
AKI etiology, n (%)				
Ischemic	25 (24.5)	10 (25.0)	15 (24.2)	0.84
Drug-induced	40 (42.2)	14 (35.0)	26 (41.9)	0.22
Glomerulonephritis	29 (28.4)	11 (27.5)	18 (29.0)	0.84
Sepsis	4 (3.9)	3 (7.5)	1 (1.6)	0.14
Others	4 (3.9)	2 (5.0)	2 (3.2)	0.77
AKI stage 2&3, n (%)	80 (78.4)	35 (87.5)	45 (72.6)	0.07
RRT, n (%)	46 (47.1)	22 (55.0)	24 (38.7)	0.04
Pathology diagnosis				
ATI, tubulointerstitial involvement (%)				
- < 25%	62 (60.8)	17 (42.5)	45 (72.6)	0.002
- 25% to 50%	24 (23.5)	13 (32.5)	11 (17.7)	0.06
- > 50%	16 (15.7)	10 (25.0)	6 (9.7)	0.04
Other lesions combined with ATI, n (%)				
Malignant arteriolar sclerosis	3 (2.9)	3 (7.5)	0 (0)	0.08
TMA	3 (2.9)	1 (2.5)	2 (3.2)	0.47
Glomerulonephritis	29 (28.4)	11 (27.5)	18 (29.0)	0.84
-Acute glomerulonephritis	3 (2.9)	1 (2.5)	2 (3.2)	0.47
-Crescentic glomerulonephritis	10 (9.8)	5 (12.5)	5 (8.1)	0.64
-Other glomerulonephritis	16 (15.7)	5 (12.5)	11 (17.7)	0.38
TBM rupture/TEC necrosis, n (%)	24 (23.5)	13 (32.5)	11 (17.7)	0.03
Parameters at time of AKI diagnosis				
Serum creatinine, µmol/L	441.8 ± 31.6	527.4 ± 54.8	386.5 ± 36.9	0.02
Serum albumin, g/L	33.0 ± 1.9	33.2 ± 2.3	32.5 ± 1.7	0.80
Hemoglobin, g/L	109.2 ± 3.4	95.6 ± 4.2	117.7 ± 4.4	0.001
Plasma CK20, ng/mL	3.4 (2.0-6.1)	4.3 (2.3-6.9)	3.1 (1.9-5.9)	0.18
Urinary CK20, µg/g Cr	4.9 (2.6-9.0)	8.0 (5.1-13.6)	3.3 (1.9-5.9)	<0.001
Urinary KIM-1, µg/g Cr	3.9 (2.3-6.9)	3.8 (2.1-8.3)	4.0 (2.5-5.9)	0.86
Urinary NGAL, µg/g Cr	191 (77-615)	364 (115-1118)	138 (58-430)	0.002
Urinary TIMP-2*IGFBP-7, (µg/g Cr)^2	1443 (366-4634)	2481 (567-8096)	1203 (324-2684)	0.02
UACR, mg/g Cr	585 (88-3602)	1317 (153-4560)	404 (73-2752)	0.04

[A]Outcome was AKI to CKD or advanced CKD progression. In patients with preadmission eGFR ≥ 60 ml/min /1.73m^2, AKI to CKD progression was defined as a persistent eGFR < 60ml/min/1.73m^2 and with a minimum 25% reduction from baseline eGFR 90 days after AKI. In patients with prehospitalization eGFR 45-60ml/min/1.73m^2, AKI to advanced CKD was defined by a sustained reduction of eGFR < 30 ml/min/1.73m^2 90 days after AKI.

RRT, renal replacement therapy; ATI, acute tubular injury; TMA, thrombotic microangiopathy; TBM, tubular basement membrane; TEC, tubular epithelial cell; CK20, cytokeratin 20; NGAL, Neutrophil gelatinase-associated lipocalin; KIM-1, kidney injury molecule-1; TIMP-2, tissue inhibitor of metallopeptidase-2; IGFBP-7, insulin-like growth factor-binding protein-7; UACR, urine albumin to creatinine ratio.

Supplemental Table 3. Multivariate logistic regression analysis of uCK20 as a predictor for severe ATI[A] in 102 patients who received kidney biopsy

uCK20 (μg/g Cr) on baseline	n	Event n (%)	Unadjusted OR (95%CI)	*P* value	Adjusted OR[B] (95%CI)	*P* value
Categorical analysis						
Tertile1 (< 3.3 μg/g Cr)	34	2 (5.9)	referent		referent	
Tertile2 (3.4-7.5 μg/g Cr)	35	17 (48.6)	26.9 (3.3-219.7)	<0.001	27.2 (3.2-227.5)	0.002
Tertile3 (> 7.5 μg/g Cr)	33	21 (63.6)	66.9 (8.1-555.2)	<0.001	89.1 (9.8-809.8)	<0.001
Continuous analysis						
Per SD increase (Log10 transformed)	102	40 (39.2)	4.6 (2.1-9.9)	<0.001	5.9 (2.6-12.6)	<0.001

[A]Severe ATI was defined as injured tubular involvement > 25% in the biopsy samples.

[B]Adjusted for age, sex, serum creatinine at time of AKI diagnosis, UACR at time of AKI diagnosis, and AKI stage.

Supplemental Table 4. Performances of each biomarker at optimal cutoff based on the Youden index

Marker	Optimal cutoff	Sensitivity	Specificity
**Test set**			
uCK20, μg/g Cr	5.0	0.80	0.75
uNGAL, μg/g Cr	274.8	0.70	0.70
uTIMP-2*IGFBP-7, $(\mu g/g\ Cr)^2$	491.6	0.82	0.45
uKIM-1, μg/g Cr	5.5	0.43	0.78
UACR, mg/g Cr	89.7	0.89	0.48
**Validation set**			
uCK20, μg/g Cr	4.1	0.78	0.67
uNGAL, μg/g Cr	234.2	0.68	0.79
uTIMP-2*IGFBP-7, $(\mu g/g\ Cr)^2$	750.8	0.80	0.60
uKIM-1, μg/g Cr	4.4	0.50	0.78
UACR, mg/g Cr	381.1	0.77	0.54

Supplemental Table 5. List of antibodies and ELISA kits used in the study

Name	Clone/label	Host	Application	Supplier
**Antibodies**				
α-CK20	monoclonal	Mouse	IHC, IF	H00054474-M01, Abnova, Taiwan
α-CK20	monoclonal	Rabbit	IHC, WB	82428-1-RR, Proteintech, Hubei
α-AQP1	monoclonal	Rabbit	IF	ab168387, Abcam, Cambridge, MA
α-UMOD	monoclonal	Rabbit	IF	ab207170, Abcam, Cambridge, MA
α-AQP2	monoclonal	Rabbit	IF	ab199975, Abcam, Cambridge, MA
α-ACSL4	monoclonal	Rabbit	IF	ab155282, Abcam, Cambridge, MA
α-pMLKL	monoclonal	Rabbit	IF	bsm-54104R, Bioss, Beijing
α-CK7	monoclonal	Mouse	IHC	66483-1-Ig, Proteintech, Hubei
α-CK7	monoclonal	Rabbit	IHC	ab181598, Abcam, Cambridge, MA
α-CK8	monoclonal	Mouse	IHC	bsm-33061M, Bioss, Beijing
α-CK8	monoclonal	Rabbit	IHC	ab53280, Abcam, Cambridge, MA
α-CK18	monoclonal	Mouse	IHC	66187-1-Ig, Proteintech, Hubei
α-CK18	monoclonal	Rabbit	IHC	ab181597, Abcam, Cambridge, MA
α-CK19	monoclonal	Mouse	IHC	60187-1-Ig, Proteintech, Hubei
α-CK19	monoclonal	Rabbit	IHC	ab52625, Abcam, Cambridge, MA
α-GAPDH	polyclonal	Rabbit	WB	10494-1-AP, Proteintech, Hubei
α-Rabbit IgG	HRP	Goat	WB	7074, Cell Signaling Technology, Beverly, MA
α-Mouse IgG	Alexa Flour® 555	Goat	IF	ab150118, Abcam, Cambridge, MA
α-Rabbit IgG	Alexa Flour® 488	Goat	IF	ab150081, Abcam, Cambridge, MA
**ELISA kits**				
CK20			ELISA	CSB-E11710h, Cusabio, Hubei
NGAL			ELISA	KIT036, Bioporto, Hellerup
KIM-1			ELISA	DY1750B, R&D Systems, Minneapolis, MN
TIMP-2			ELISA	DTM200, R&D Systems, Minneapolis, MN
IGFBP-7			ELISA	DY1334-05, R&D Systems, Minneapolis, MN

CK, cytokeratin; AQP, aquaporin; UMOD, uromodulin; ACSL4, Acyl-CoA synthetase long-chain family member 4; pMLKL, phosphorylated mixed lineage kinase domain-like protein; GAPDH, glyceraldehyde 3-phosphate dehydrogenase; HRP, horseradish peroxidase; NGAL, neutrophil gelatinase-associated lipocalin; KIM-1, kidney injury molecule-1; TIMP-2, tissue inhibitor of metallopeptidase 2; IGFBP-7, insulin-like growth factor-binding protein 7; IHC, immunohistochemistry; IF, immunofluorescence; WB, western blotting; ELISA, Enzyme-linked immunosorbent assay.
