# Supplementary material for: Urinary cytokeratin 20 as a predictor for chronic kidney disease following acute kidney injury
Source: JCI Insight. 2024 May 28;9(13):e180326. doi: 10.1172/jci.insight.180326 (PMC11383368; doi:10.1172/jci.insight.180326)
Supplement: Unedited blot and gel images [file jciinsight-9-180326-s063.pdf]

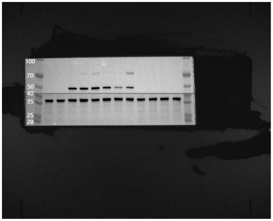

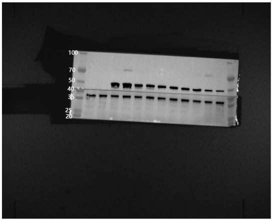

Full unedited gel for Figure 2C

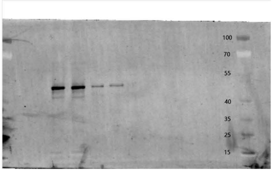

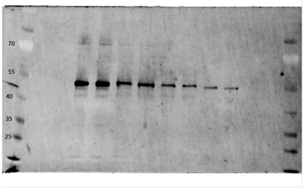

Full unedited gel for Figure 5A

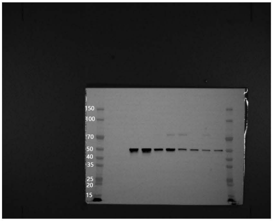

Full unedited gel for Figure 5C

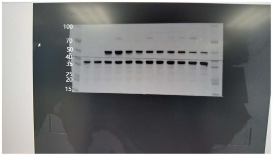

Full unedited gel for Figure S2C

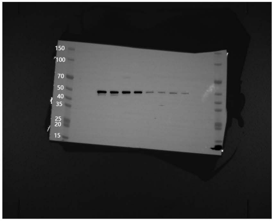

Full unedited gel for Figure S2E
